# Modulation of Increased mGluR1 Signaling by RGS8 Protects Purkinje Cells From Dendritic Reduction and Could Be a Common Mechanism in Diverse Forms of Spinocerebellar Ataxia

**DOI:** 10.3389/fcell.2020.569889

**Published:** 2021-01-21

**Authors:** Qin-Wei Wu, Josef P. Kapfhammer

**Affiliations:** Institute of Anatomy, Department of Biomedicine, University of Basel, Basel, Switzerland

**Keywords:** RGS8, mGluR1, Purkinje cells, SCA14, spinocerebellar ataxias

## Abstract

Spinocerebellar ataxias (SCAs) are a group of hereditary neurodegenerative diseases which are caused by diverse genetic mutations in a variety of different genes. We have identified RGS8, a regulator of G-protein signaling, as one of the genes which are dysregulated in different mouse models of SCA (e.g., SCA1, SCA2, SCA7, and SCA14). In the moment, little is known about the role of RGS8 for pathogenesis of spinocerebellar ataxia. We have studied the expression of RGS8 in the cerebellum in more detail and show that it is specifically expressed in mouse cerebellar Purkinje cells. In a mouse model of SCA14 with increased PKCγ activity, RGS8 expression was also increased. RGS8 overexpression could partially counteract the negative effects of DHPG-induced mGluR1 signaling for the expansion of Purkinje cell dendrites. Our results suggest that the increased expression of RGS8 is an important mediator of mGluR1 pathway dysregulation in Purkinje cells. These findings provide new insights in the role of RGS8 and mGluR1 signaling in Purkinje cells and for the pathology of SCAs.

## Introduction

Spinocerebellar ataxias (SCAs) are a heterogeneous group of progressive genetic disorders with degeneration and dysfunction of the cerebellum (Chen et al., 2005; Gatchel et al., 2008; Dansithong et al., 2015; Klockgether et al., 2019). The genetic background of SCAs can be classified into two groups: Group I, repeat expansion SCAs, are caused by dynamic repeat expansion mutations, such as SCA1 and SCA2, and Group II, conventional mutation SCAs (non-repeat expansion SCAs), are caused by mutations, deletions or insertions in specific genes, such as in beta-III Spectrin in SCA5 or in Protein Kinase C gamma (PKCγ) in SCA14 (Klockgether et al., 2019). Most SCA mutations cause cerebellar damage and dysfunction typically resulting from Purkinje cell degeneration (Chen et al., 2005; Klockgether et al., 2019).

Due to the diversity of the affected genes, it is not clear in the moment whether there is a single disease mechanism causing the diverse forms. However, transcriptional changes in molecules that mediate the development of Purkinje cells are a hallmark of SCAs and determine the severity of the disease (Serra et al., 2006). The identification of molecules with transcriptional changes in different SCAs could reveal molecular mechanisms underlying the pathogenesis of SCAs. Previously, SCA1 and SCA7 mouse models have been chosen as the representatives of group I repeat expansion SCAs, and 27 molecules with transcriptional changes in both mouse models have been identified by using a microarray-based gene profiling strategy (Gatchel et al., 2008).

In the current study, we have used microarray data from an SCA14 mouse model (Shimobayashi et al., 2016) as a representative of Group II SCAs, and compared them with the common genes found in SCA1 and SCA7 (Gatchel et al., 2008) and genes found to be dysregulated in SCA2 (Dansithong et al., 2015). This approach identified RGS8 as the only molecule which was dysregulated in SCA1, SCA2, SCA7, and SCA14 mouse models. We studied the changes of RGS8 in the SCA14 mouse model in more detail and found that upregulation of RGS8 is associated with increased mGluR1 signaling. In addition, we present evidence suggesting that elevated RGS8 may act as a protective mediator of increased mGluR1 signaling.

## Materials and Methods

### Animals

All experiments were carried out in accordance with the EU Directive 2010/63/EU for the care and use of laboratory animals, were approved by the veterinary office of the canton of Basel and permitted by Swiss authorities. FVB mice were used for primary cerebellar cell cultures. PKCγ knockout (KO) mice were constructed and generated by CRISPR/Cas9-mediated gene editing technology in the Centre for Transgenic Models, University of Basel. The SCA14 conditional transgenic mice with FVB background used in this study have been described previously (Trzesniewski et al., 2019). Briefly, in order to generate a conditional transgenic mouse line to express the SCA14 associated human PKCγ(S361G) mutation specifically in Purkinje cells, transgenic mice including PKCγ(S361G) with tetracycline response element (TRE) and green fluorescent protein (GFP) reporter were crossed with Pcp2-tTA transgenic mice which express the Tet-Transactivator under a Purkinje cell specific promoter.

### Organotypic Slice Cultures

Organotypic slice cultures were made as described previously (Kapfhammer and Gugger, 2012). Mice were decapitated at postnatal day 8, and their brains were aseptically dissected. The cerebellum was separated in ice-cold minimal essential medium (MEM) supplemented with 1% glutamax (Gibco, Invitrogen) and sagittal slices of 350 μm thickness were cut with a McIlwain tissue chopper under sterile conditions. Cerebellar slices were separated, transferred onto a permeable membrane (Millicell-CM, Millipore), and incubated with incubation medium (50% MEM, 25% Basal Medium Eagle, 25% horse serum, 1% glutamax, 0.65% glucose) or Neurobasal medium (97% Neurobasal medium, 2% B27, 1% glutamax) under 5% CO_2_ at 37°C. The medium was refreshed every 2 or 3 days.

### Immunostainings

Immunohistochemistry was performed as described previously (Trzesniewski et al., 2019). For cerebellar sections, mice were sacrificed and perfused with 4% paraformaldehyde. Sagittal sections were cut with a cryostat (Leica) at 20 μm. Organotypic slice cultures were fixed after 7 days *in vitro* in 4% paraformaldehyde for 6–24 h at 4°C. Primary dissociated cerebellar cultures were fixed in 4% paraformaldehyde for 30 min at room temperature. All reagents were diluted in 100 mM phosphate buffer (PB). The sections or slices were incubated with primary antibody diluted in blocking solution (PB + 3% non-immune goat serum + 0.3% TritonX-100) overnight at 4°C, dissociated cerebellar cultures for 1 h at room temperature. After washing with PB, the corresponding fluorescence-conjugated secondary antibodies were added to the slices in PB containing 0.1% Triton X-100 for 2 h at room temperature. The following primary antibodies were used: rabbit anti-Calbindin D-28K (1:500, Swant, Marly, Switzerland); mouse anti-Calbindin D-28K (1:500, Swant, Marly, Switzerland); sheep anti-RGS8 (1:100, R&D Systems). The following secondary antibodies were used: goat anti-mouse Alexa 488 (1:500, Molecular Probes, Invitrogen); goat anti-rabbit Alexa 488 (1:500, Molecular Probes, Invitrogen); donkey anti-sheep Alexa 568 (1:500, Molecular Probes, Invitrogen). Stained slices or sections were mounted with Mowiol (Sigma-Aldrich, Buchs, Switzerland). The images were captured with an Olympus AX-70 microscope equipped with a Spot Insight digital camera or a Zeiss LSM700 upright confocal microscope.

### Western Blot and Immunoprecipitation

Cerebellar slices were lysed with RIPA buffer in the presence of protease and phosphatase inhibitors. Samples were separated by SDS-PAGE and blotted on a nitrocellulose membrane. After blotting, membranes were incubated with 5% BSA in TBS for 1 h and incubated with the specific primary antibodies. After washing with TBS-T, membranes were incubated with HRP-labeled secondary antibodies. Proteins were visualized by ECL (Pierce, Thermo Fisher Scientific, Reinach, Switzerland). Alternatively, membranes were incubated with IRDye^®^ Secondary Antibodies for 1 h. The proteins were quantified using C-Digit Western Blot software (LI-COR Biosciences, Bad Homburg, Germany). HEK293T cells were transfected with plasmids pCMV-mGluR1, pCMV-PKCγ-tGFP or pCMV-PKCγ(S361G)-tGFP using Lipofectamine 3000 (Invitrogen) according to manufacturer’s instructions and incubated for 24–48 h before harvest. tGFP trap agarose beads (Chromotek) were used for immunoprecipitation of tGFP-labeled PKCγ(S361G) or WT PKCγ proteins according to manufacturer’s instructions. The following primary antibodies were used in this study: sheep anti-RGS8 (1:1000, R&D Systems), mouse anti-actin (1:2000, Millipore), rabbit anti-alpha Tubulin (1:1000, Proteintech), rabbit anti-phospho-PKC substrate (1:1000, Cell Signaling), mouse anti-GAPDH (1:4000, Proteintech); rabbit anti-mGluR1 (1:1000, Cell Signaling); rabbit anti-mGluR5 (1:1000, Abcam); mouse anti-Gαq/11 (1:300, Santa Cruz); mouse anti-turboGFP (1:1000, Origene); rabbit anti-turboGFP (1:1000, Invitrogen); mouse anti-Myc (1:1000, Origene). The following secondary antibodies were used in this study: anti-sheep HRP conjugate antibody (1:1000, R&D Systems); anti-mouse HRP conjugate antibody (1:10,000, Promega); anti-rabbit HRP conjugate antibody (1:10,000, Promega); IRDye^®^ 680LT Goat anti-Rabbit IgG Secondary Antibody (1:10,000, LICOR); IRDye^®^ 800CW Goat anti-Mouse IgG Secondary Antibody (1:10,000, LICOR).

### Microarray Study and Quantitative Real-Time Polymerase Chain Reaction

The data of genes whose expression is commonly altered in SCA1 and SCA7 mouse models was used from published data (Gatchel et al., 2008). The data of top 50 genes in SCA2 mouse models was used from the study of Dansithong et al. (2015). The data from organotypic cerebellar slice cultures of SCA14 PKCγ(S361G) and control mice were previously established in the laboratory (Shimobayashi et al., 2016).

### Plasmid Construction

pCMV-Rgs8 and pCMV-mGluR1 vectors were from Origene (Rockville, MD, United States). Plasmid L7-GFP was previously described and a gift from Dr. Wolfgang Wagner (2011). Linearized pL7 vectors were produced by the restriction enzymes NotI and NcoI (New England BioLabs, Ipswich, MA, United States) and mouse *Rgs8* gene insert fragments were generated by polymerase chain reaction (PCR). pL7-Rgs8-GFP were produced by In-Fusion HD Cloning Kits (Clontech, Mountain View, CA, United States). The following primers were used for PCR: Rgs8 forward CAG GAT CCA GCG GCC GCA TGG CTG CCT TAC TGA TGC CA; Rgs8 Reverse CCC TTG CTC ACC ATG GTG CTG AGC CTC CTC TGG CTC TG. pCMV-PKCγ-tGFP, pCMV-PKCγ(S361G)-tGFP and pL7-PKCγ(S361G)-GFP were gifts from Dr. Etsuko Shimobayashi (Shimobayashi and Kapfhammer, 2017).

### Primary Cerebellar Cell Cultures and Transfection

Primary cerebellar cell cultures were prepared from neonatal mice as described previously (Wagner et al., 2011; Shimobayashi and Kapfhammer, 2017). Briefly, cerebella from postnatal day 0 mice were dissected, dissociated and plated on glass chambers coated with Poly-D-lysine. Indicated vectors were introduced into Purkinje cells by transfection with a Neon Transfection System (Thermo Fisher Scientific) using the following settings: Pulse voltage 1,200 V, Pulse width 30 ms, Pulse number 1. Cells were incubated in incubation medium (90% Dulbecco’s modified Eagle medium/F-12 nutrient-based medium, 1% N2 supplement, 1% glutamax, and 10% FBS). 2–4 h after transfection, 500 μl DFM supplemented 1% N2 and 1% glutamax was added to each well. After that, half of the medium was refreshed every 4 days. The media and supplements were from Life Technologies, Zug, Switzerland. Cells were kept in culture for 2 weeks before fixation. The following pharmacological compounds were added to the medium at 7 days *in vitro*: (S)-3,5-Dihydroxyphenylglycine hydrate (DHPG) (Sigma-Aldrich, St. Louis, MO, United States).

### Quantification of Purkinje Cell Dendritic Expansion and Fluorescence Intensity of Immunostainings in Dissociated Cerebellar Cultures

The quantification of Purkinje cell dendritic tree size was performed as previously described (Shimobayashi and Kapfhammer, 2017). The average value of control Purkinje cells was set as 1. In order to ensure a comparable growth environment, non-transfected Purkinje cells close to the Purkinje cells transfected with the indicated vectors from the same well were taken as control in this study. An image analysis program (ImageJ) was used to trace the outline of the Purkinje cell dendritic trees yielding the area covered by the dendritic tree. The mean fluorescence intensity of the Purkinje cell soma was calculated by ImageJ and the raw images were used for the fluorescence intensity analysis. The shown images were linearly adjusted in brightness and contrast. The data were analyzed using GraphPad Prism software (San Diego, CA, United States). The statistical significance of differences in parameters was assessed by the nonparametric two-tailed Mann–Whitney’s test. Confidence intervals were 95%, statistical significance was assumed with *P* < 0.05.

## Results

### RGS8 Is Dysregulated in Several SCAs

**FIGURE 1:**
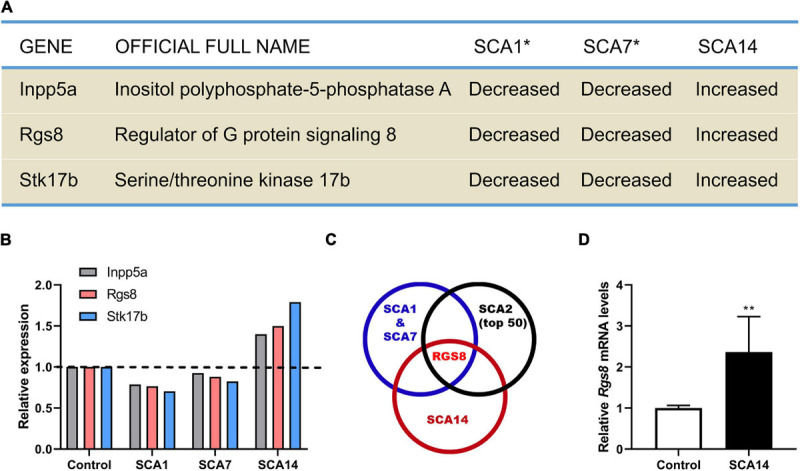
Identification RGS8 as a molecule with dysregulated expression in different SCA mouse models. **(A)** Dysregulated genes in SCA1, SCA7, and SCA14 mouse models. Asterisk indicates that the data of SCA1 and SCA7 from the published microarray data sets (Gatchel et al., 2008). **(B)** The relative transcriptional levels of Inpp5a, Rgs8, and Stk17b in SCA1, SCA7, and SCA14 were evaluated by microarrays. The relative changes of Inpp5a are 0.787, 0.926, and 1.399, respectively. The relative changes of Rgs8 are 0.766, 0.88, and 1.499, respectively. The relative changes of Stk17b are 0.704, 0.825, and 1.791, respectively. The data of SCA1 and SCA7 were obtained from the published microarray data sets (Gatchel et al., 2008). **(C)** Comparison of the common dysregulated genes in SCA1, SCA7, and SCA14 with the top 50 altered genes in SCA2 mice. The data of SCA2 and the common genes of SCA1 and SCA7 were obtained from published studies (Gatchel et al., 2008; Dansithong et al., 2015). RGS8 is the only dysregulated gene in all 4 SCA mouse models. **(D)** Relative expression of Rgs8 in SCA14 evaluated by real-time qPCR. GAPDH was used as a housekeeping gene. The mean value of control is 1.000 ± 0.0659; the mean of SCA14 is 2.364 ± 0.8633. Two biological replicates were done in triplicate. Data are expressed as mean ± SD. **P < 0.01 was determined by the two-tailed Mann–Whitney test.

By comparing the transcriptional expression of various genes in SCA1 and SCA7 mouse models 27 common genes have been identified which were suggested to be involved in the pathogenesis of SCAs (Gatchel et al., 2008). We have now further compared these genes to transcriptional changes in a mouse model of SCA14 (Ji et al., 2014; Shimobayashi et al., 2016), and have identified three genes which were dysregulated in all three mouse models. Interestingly, these three molecules were strongly up-regulated in the SCA14 model but down-regulated in SCA1 and SCA7 models (Figures 1A,B).

Inpp5a has already been shown to play a crucial role for the survival of Purkinje cells (Yang et al., 2015; Liu et al., 2020). Stk17b, also known as Drak2, is strongly expressed in lymphoid organs and known to transmit non-apoptotic signals during thymocyte differentiation (Friedrich et al., 2005). We further compared the key molecules to the top 50 changed transcripts in the SCA2 mouse model (Dansithong et al., 2015) and found that RGS8 is the unique dysregulated gene in the four different SCA mouse models (Figure 1C), with a reduced expression on SCA1, 2, and 7, but an increased expression in SCA14. In order to confirm the upregulation in the SCA14 mouse model, we performed qPCR which confirmed *Rgs8* upregulation at mRNA level (Figure 1D). These results demonstrate that RGS8 is unique in being a dysregulated molecule in at least 4 SCA mouse models.

### RGS8 Is Expressed in Purkinje Cells Starting at Early Postnatal Development

**FIGURE 2:**
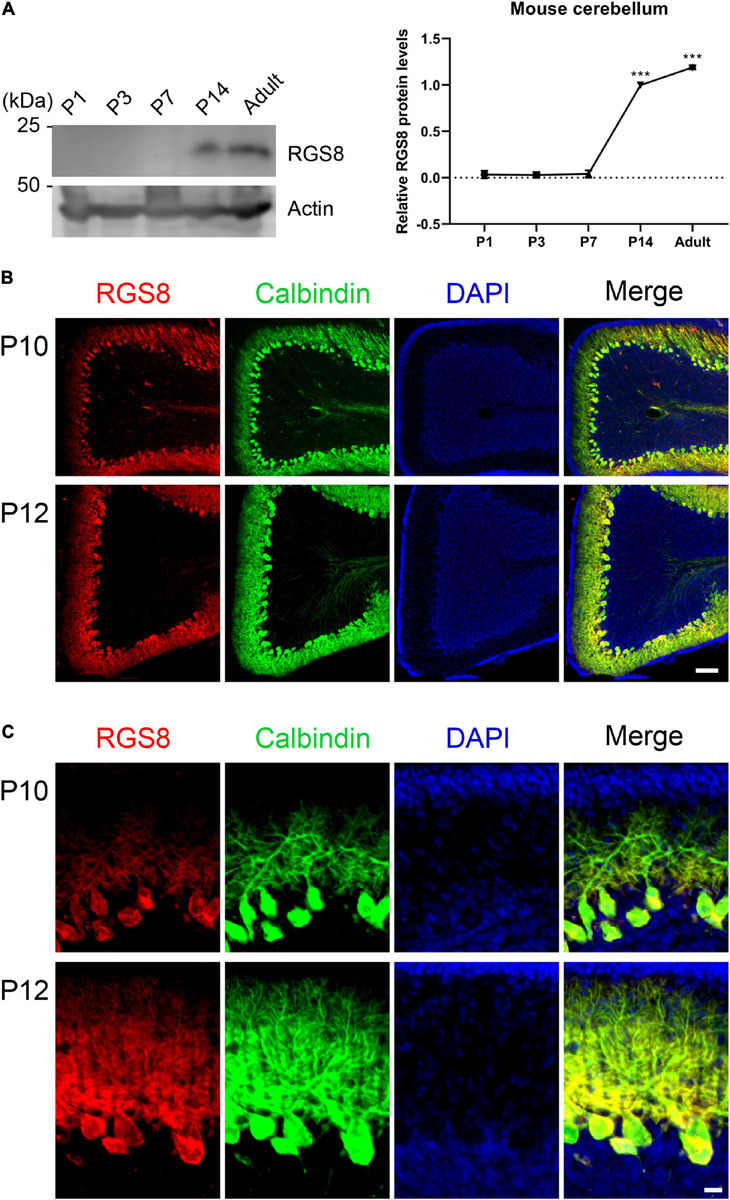
Expression of RGS8 protein in the postnatal mouse cerebellum. **(A)** Western blots from mouse cerebellum at different postnatal stages. Left side: RGS8 expression is first evident at P14. Actin used as loading control shown in bottom panel. Right side: Quantification of protein levels from Western blots. Data are expressed as mean ± SD, with three independent biological samples for P1 (three or four pups for one sample), P3 (three or four pups for one sample) and P7, and two independent biological samples for P14 and Adult. The mean value of RGS8 protein level at P14 was set as 1, and values for the other stages were expressed relatives of this value. The mean values of P1, P3, P7 and adult were 0.034 ± 0.0410, 0.029 ± 0.0179, 0.041 ± 0.0386, and 1.191 ± 0.0217. ***P < 0.001 was determined by one-way ANOVA with multiple comparisons (P14 vs. P1, P3, and P7; Adult vs. P1, P3, P7, and P14). **(B)** RGS8 immunoreactivity (red signal) is present in cerebellar Purkinje cells (identified by anti-Calbindin, green) at P10 and P12. Scale bar is 100 μm. **(C)** Viewed at higher magnification, RGS8 is present in dendrites and the soma of Purkinje cells at P10 and P12. Scale bar is 20 μm.

The temporal expression profile of RGS8 was investigated in the developing cerebellum of the mouse from postnatal day 1 (P1) to adult by Western blot. No signal on Western blots of P1–P7 suggested that RGS8 is not or only weakly expressed in cerebellar cells during the first postnatal week. It was mainly detectable in mouse cerebellum after P7 and remained expressed through adulthood (Figure 2A). In order to confirm that RGS8 was expressed in Purkinje cells, sagittal cerebellar sections were collected from the mouse cerebellum at P10 and P12. Purkinje cells, which were identified by labeling with the Purkinje cell marker Calbindin, were nicely stained by the RGS8 antibody. RGS8 staining extended from the Purkinje cell layer to the Molecular layer of the cerebellum, and RGS8 immunoreactivity was present in the cell body and the dendrites of Purkinje cells at P10 and P12 (Figures 2B,C), and no immunoreactivity could be detected in Purkinje cells at P7 (Supplementary Figure 1). These data confirm that RGS8 is expressed in Purkinje cells starting in the second postnatal week suggesting that it plays a role during postnatal Purkinje cell differentiation and maturation.

### Increased RGS8 Protein Expression in Purkinje Cells of the PKCγ(S361G) Transgenic SCA14 Mouse Model

**FIGURE 3:**
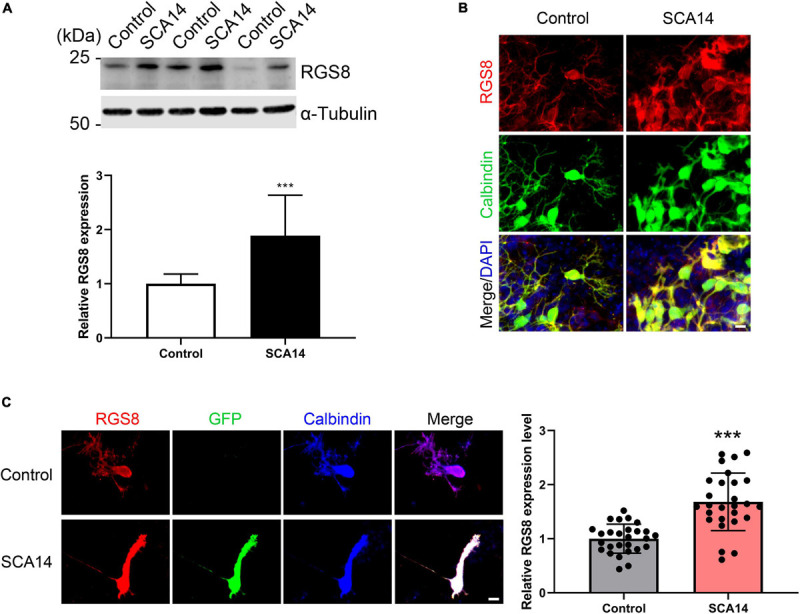
Increased RGS8 expression in Purkinje cells of the SCA14 S361G mouse model. **(A)** Western blots of protein extracts from at least three different experiments with organotypic cerebellar slice cultures from SCA14 PKCγ(S361G) and control mice. The quantification of the Western blots showed a 1.89 ± 0.749 fold increased expression in slice cultures from SCA14 PKCγ(S361G) transgenic mice. Data from at least three independent biological replicates. **(B)** RGS8 immunoreactivity was increased in Purkinje cells in cerebellar slice cultures from SCA14 PKCγ(S361G) mice versus littermate controls. Scale bar is 20 μm. **(C)** RGS8 immunoreactivity was increased in Purkinje cells from SCA14 PKCγ(S361G) transgenic mice in mixed dissociated cultures containing Purkinje cells derived from transgenic and control mice. Purkinje cells were identified by anti-calbindin staining (blue), and PKCγ(S361G)-transgenic cells by anti-GFP staining (green). The fluorescence of anti-RGS8 staining was quantified using ImageJ. The mean value of RGS8 expression for SCA14 PKCγ(S361G) is increased 1.68 ± 0.533 fold compared to control cells and the *n* was 27. Data are expressed as mean ± SD. ***P < 0.001 was determined by the two-tailed Mann–Whitney test. Scale bar is 20 μm. Please note that PKCγ(S361G) transgenic Purkinje cells have a changed morphology as reported earlier (Ji et al., 2014).

The gene array had indicated an increased expression of RGS8 mRNA and we now confirmed an increased expression on the protein level. We further found that RGS8 protein expression was increased in Western blots of organotypic cerebellar cultures from SCA14 PKCγ(S361G) mice (Figures 3A,B).

In mixed dissociated cultures from S361G transgenic and control mouse pups the transgenic Purkinje cells were identified by endogenous GFP expression. We then quantified RGS8 immunoreactivity on GFP-positive Purkinje cells from S361G-transgenic mice versus GFP-negative Purkinje cells from control mice present in the same culture well (Figure 3C). Purkinje cells from S361G-trangenic mice have an altered morphology with reduced and thickened dendrites as previously reported (Ji et al., 2014; Shimobayashi et al., 2016). RGS8 immunoreactivity was increased to a 1.68 ± 0.533 fold higher expression of RGS8 in Purkinje cells from S361G-transgenic mice versus GFP-negative Purkinje cells from control mice (Figure 3C) present in the same culture well.

### Increased RGS8 Expression in the PKCγ(S361G) Cerebellum Is Associated With Signs of Elevated mGluR1 Signaling

**FIGURE 4:**
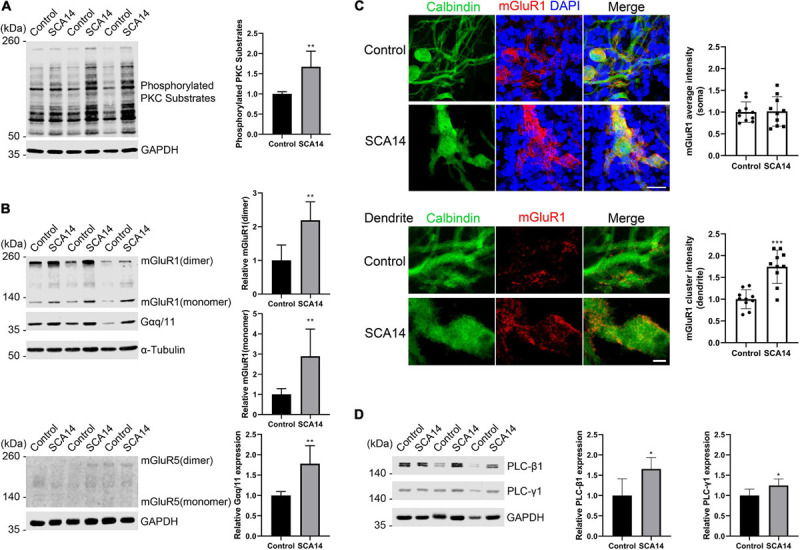
mGluR1-PKCγ signaling molecules have increased expression in the SCA14 mouse model. **(A)** Western blot of cerebellar slice cultures of PKCγ(S361G) and control mice stained for phosphorylated PKC substrates. Quantification of protein levels from Western blots. The mean value of the phosphorylated PKC substrates for control is 1.00 ± 0.053; the mean value of the phosphorylated PKC substrates for SCA14 PKCγ(S361G) is 1.67 ± 0.388. Three biological replicates. Data are expressed as mean ± SD. **(B)** mGluR1 and Gαq/11 expression in protein extracts from cerebellar slice cultures of PKCγ(S361G) and control mice, α-Tubulin is shown as loading control. The mean value of mGluR1 dimer in S361G-derived slice cultures is increased 2.19 ± 0.546 fold compared to control. The mean value of mGluR1 monomer in PKCγ(S361G)-derived slice cultures is increased 2.89 ± 1.345 fold compared to control, the mean value of Gαq/11 in PKCγ(S361G)-derived slice cultures is increased 1.78 ± 0.441 fold compared to control. No mGluR5 signal could be detected in protein extracts from cerebellar slice cultures of PKCγ(S361G) and control mice. **(C)** Top panel: staining of cerebellar slice cultures from PKCγ(S361G) and littermate control mice with anti-mGluR1 antibodies. Scale bar is 20 μm. Bottom panel: dendrites viewed at higher magnification. Scale bar is 5 μm. The mean value of the mGluR1 average intensity on soma for control is 1.00 ± 0.234; the mean value of the mGluR1 average intensity on soma for SCA14 PKCγ(S361G) is 1.02 ± 0.339, *n* = 10 cells, and the difference in expression was not significant in the two-tailed Mann–Whitney test. The mean value of the mGluR1 cluster intensity on dendrite for control is 1.00 ± 0.215; the mean value of the mGluR1 cluster intensity on dendrite for SCA14 PKCγ(S361G) is 1.75 ± 0.383, *n* = 10 cells. **(D)** Western blot analysis and their quantification of components of mGluR1 downstream signaling from cerebellar slice cultures. An increase (PLC-β1: 1.65 ± 0.278 fold; PLC-γ1: 1.25 ± 0.158 fold) in phosphoinositide phospholipase C (PLC) isozymes was observed in protein extracts from cerebellar slice cultures of SCA14 PKCγ(S361G) mouse line. Three biological replicates. Data are expressed as mean ± SD. *P < 0.05, **P < 0.01, and ***P < 0.001 were determined by the two-tailed Mann–Whitney test.

In S361G mice there is a constitutive activation of PKCγ signaling. We verified the increased phosphorylation of PKCγ target proteins by antibodies that recognize phosphorylation of PKC substrates. In extracts from cerebellar slice cultures from SCA14 PKCγ(S361G) mice there was a strong increase of target phosphorylation. The mean value was increased to 1.67 ± 0.388 fold in S361G derived organotypic slice cultures compared to cultures from littermate controls (Figure 4A).

RGS8 belongs to the R4 subfamily of RGS proteins, all of which accept Gαq/11 subunit as substrates, and the structure of RGS8-Gαq complex has been reported recently (Taylor et al., 2016; Squires et al., 2018). RGS8 can interact with Gαq/11 in brain membranes of rat (Saitoh et al., 1997). The metabotropic glutamate receptor 1 (mGluR1) is coupled to the Gαq pathway and strongly expressed in cerebellar Purkinje cells (Tanaka et al., 2000), and it has been suggested that RGS8 is associated with activation of the mGluR1-Gαq pathway in an ataxin-2 mouse model (Dansithong et al., 2015). As mGluR1 signaling is supposed to be upstream of PKCγ signaling, we wondered whether it would be synergistically increased or as a compensation decreased in slice cultures from S361G mice. We found by immunohistochemistry that mGluR1 was expressed in Purkinje cells in organotypic cultures of SCA14 mice (Supplementary Figure 2). In Western blots, two immunoreactive bands of mGluR1 were present representing monomeric and dimeric forms. mGluR1 dimer expression was increased to 2.19 ± 0.546 fold of control. mGluR1 monomer expression was strongly increased to 2.89 ± 1.345 fold of control. The associated increase of Gαq/11 was 1.78 ± 0.441 fold of control (Figure 4B). In agreement with previous studies (Shin et al., 2015), mGluR1 is the main group I mGluR in the cerebellum where it is highly expressed. We did not observe expression of mGluR5, another group I member, in the extracts from cerebellar organotypic slice cultures (Figure 4B). Confocal immunofluorescence studies showed that mGluR1 in SCA14 PKCγ(S361G) Purkinje cells had a similar distribution compared to wild-type cells, but showed increased expression. It was present in clusters on dendrites (Figure 4C), just like in wild-type Purkinje cells. Interestingly, in SCA14 PKCγ(S361G) Purkinje cells we also found increased levels of PLC isoforms, which can catalyze the hydrolysis of PIP2, an intermediate in the PKC pathway (Figure 4D). These results show that in Purkinje cells of the SCA14 PKCγ(S361G) mouse model there is an increased expression of several elements of the mGluR1 signaling pathway, suggesting that mGluR1-PKCγ signaling is increased in these Purkinje cells.

### mGluR1 Interacts With Mutant PKCγ(S361G)

**FIGURE 5:**
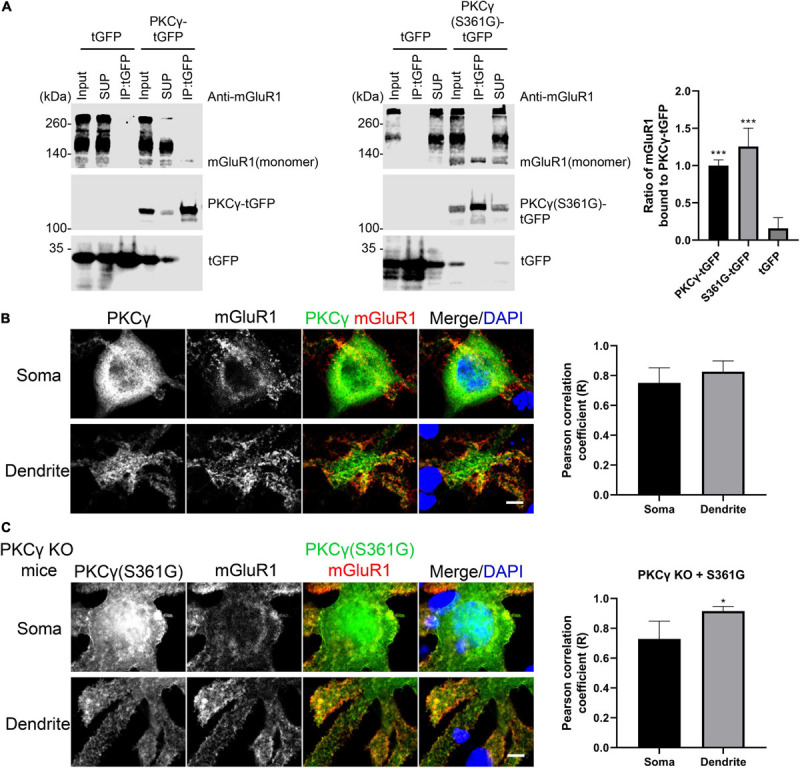
Interaction of mGluR1 and mutated PKCγ(S361G). **(A)** HEK293T cells expressing mGluR1 were transiently transfected with either PKCγ(S361G)-tGFP or tGFP alone. Anti-tGFP immunoprecipitates (IPs), inputs, and supernatants (SUP) were analyzed by Western blot with anti-mGluR1 and Anti-tGFP antibodies. Relative amount of PKCγ(S361G)-tGFP(1.26 ± 0.245) or tGFP(0.16 ± 0.143) alone bound to mGluR1 and normalized to wild-type PKCγ-tGFP(1.00 ± 0.076) bound to mGluR1. Data are expressed as mean ± SD. ***P < 0.001 was determined by the one-way ANOVA with multiple comparisons (PKCγ-tGFP vs. tGFP; S361G-tGFP vs. tGFP) and the difference between S361G-tGFP and PKCγ-tGFP was not significant. Western blots were performed three times with similar results. **(B)** Colocalization of mGluR1 and PKCγ in Purkinje cells from wild-type mice. Right shows quantification of colocalization of PKCγ with mGluR1 in soma and dendrite by the Pearson correlation coefficient (R). Soma: 0.75 ± 0.1005; Dendrite: 0.83 ± 0.0727. The *n* was 6 cells and *P* = 0.2403 in the two-tailed Mann–Whitney test. **(C)** Colocalization of mGluR1 and PKCγ(S361G) in Purkinje cells from PKCγ knockout mice transfected with mutated PKCγ(S361G). Right shows quantification of colocalization of mutated PKCγ(S361G) with mGluR1 in soma and dendrite by the Pearson correlation coefficient (R). Soma: 0.73 ± 0.1196; Dendrite: 0.93 ± 0.0321. The *n* was 6 cells and *P* = 0.0195 (*P < 0.05) in the two-tailed Mann–Whitney test. Data are expressed as mean ± SD. Scale bars in panels **(B)** and **(C)** are 5 μm.

Activated PKCγ phosphorylates target proteins involved in diverse cellular signaling pathways and has been shown to phosphorylate mGluR1 mediating signaling in cerebellar Purkinje cells (Mao et al., 2008; Kato et al., 2012). We tested whether the mutated PKCγ protein from the PKCγ(S361G) mutant mouse can interact with mGluR1. Immunoprecipitation studies with proteins from transfected HEK293T cells confirmed that PKCγ(S361G) interacts with mGluR1 (Figure 5A). Colocalization of mGluR1 and wild-type PKCγ was confirmed in Purkinje cells (Figure 5B).

Because wild-type PKCγ is co-expressed together with mutated PKCγ(S361G) in the transgenic mice, we used PKCγ knockout mice in order to confirm the interaction of mutated PKCγ protein and mGluR1 in Purkinje cells. When PKCγ(S361G) was transfected into Purkinje cells from PKCγ knockout mice, we still observed the colocalization of PKCγ(S361G) and mGluR1, in particular at the plasma membrane and on the surface of the dendrites (Figure 5C). Our results indicate that the mutant PKCγ(S361G) with constitutively catalytic activity interacts with mGluR1 and is likely to induce increased mGluR1 signaling.

### RGS8 Upregulation Counteracts the Negative Effect of DHPG-Induced mGluR1 Activation in Purkinje Cells

**FIGURE 6:**
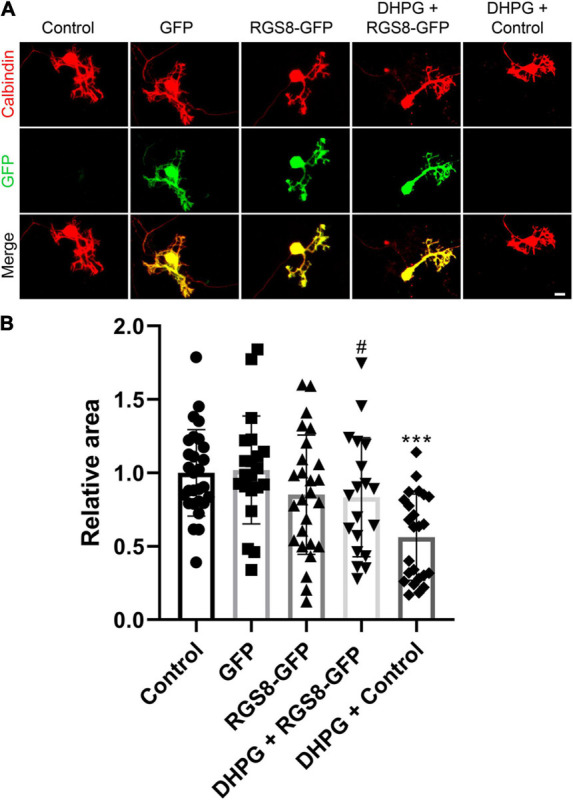
RGS8 protects the Purkinje cell dendritic tree from overshooting mGluR1 signaling. **(A)** Representative images of Purkinje cells transfected with the indicated plasmids or treated with the indicated condition. **(B)** The mean values of the Purkinje cell dendritic area were measured in at least three independent culture wells, controls were the non-transfected cells from the same culture well. Control: 1.00 ± 0.29, *n* = 28 cells; GFP transfection: 1.02 ± 0.37, *n* = 21 cells; RGS8-GFP transfection: 0.85 ± 0.41, *n* = 26 cells; RGS8-GFP with DHPG treatment: 0.83 ± 0.40, *n* = 19 cells; Control cells with DHPG treatment: 0.56 ± 0.29, *n* = 24 cells. Control vs. GFP, *P* = 0.8020; Control vs. RGS8-GFP, *P* = 0.1455; GFP vs. RGS8-GFP, *P* = 0.1101; RGS8-GFP vs. DHPG + RGS8-GFP, *P* = 0.8547; DHPG + RGS8-GFP vs. DHPG + Control, *P* = 0.0184 (^#^*P* < 0.05); DHPG + Control vs. Control, *P* < 0.0001 (***P < 0.001) in one-way ANOVA with Kruskal–Wallis test. RGS8 transfection rescues the Purkinje cell dendritic tree from reduction by 2.5 μM DHPG treatment. Data are expressed as mean ± SD. Scale bar is 20 μm.

To explore the function of RGS8 in Purkinje cells, we transfected a RGS8-GFP fusion protein in Purkinje cells using a vector with the Purkinje cell specific L7 promoter (Wagner et al., 2011; Shimobayashi et al., 2016). The dendritic expansion of Purkinje cells transfected with RGS8-GFP showed a trend toward decreasing dendritic tree size compared to non-transfected Purkinje cells in same culture well or Purkinje cells transfected with GFP tag alone, but the reduction did not reach statistical significance. Transfection of the GFP tag protein alone did not affect morphology of Purkinje cells compared to non-transfected Purkinje cells in the same culture well (Figures 6A,B). We then tested the effect of RGS8 overexpression on the mGluR1 signaling pathway. Treatment with the selective mGluR1 agonist, (S)-3,5-Dihydroxyphenylglycine (DHPG), strongly decreased the dendritic area of Purkinje cells compared to control in dissociated cultures (Figures 6A,B) in agreement with previous studies (Sirzen-Zelenskaya et al., 2006; Gugger and Kapfhammer, 2010). When Purkinje cells in dissociated cultures overexpressed RGS8-GFP, treatment with DHPG did not significantly change the dendritic area of overexpressing Purkinje cells showing that RGS8-GFP expression rescued the morphology of Purkinje cells after DHPG treatment and increased the size of the dendritic tree compared to control Purkinje cells treated with DHPG (Figures 6A,B). This finding is direct evidence for an important role of RGS8 with respect to increased mGluR1 signaling. We also tested whether RGS8 could rescue the altered dendritic morphology of PKCγ(S361G) Purkinje cells by transfection and overexpression of these cells with RGS8 (Supplementary Figure 3) and found no evidence for a protection. This could be due to the fact that RGS8 expression is already very high in PKCγ(S361G) Purkinje cells and a further increase would not be effective. It also shows that the dendritic reduction in the PKCγ(S361G) Purkinje cells is not only due to the effect of DHPG-induced mGluR1 activation and must involve additional mechanisms. Our findings show that RGS8 has an inhibitory role for the mGluR1 signaling pathway in Purkinje cells.

## Discussion

Based on its transcriptional dysregulation in four different types of SCA, we have identified RGS8 as a potential molecule involved in the pathology of SCAs. RGS8 is specifically expressed in Purkinje cells from early postnatal development. In the SCA14 PKCγ(S361G) mouse model studied in our laboratory, RGS8 expression was strongly increased in organotypic slice cultures in parallel with a reduction of dendritic tree size of the Purkinje cells. While in this mouse model the mutation produces a constitutively active form of PKCγ, we have shown that mutated PKCγ binds to mGluR1 and that there is also a strong elevation of mGluR1 expression linking SCA14 to changes in mGluR1 signaling. RGS8 is thought to negatively regulate G-protein mediated signaling. We could show that RGS8 overexpression can indeed protect Purkinje cells from the negative effects of mGluR1 activation on dendritic growth suggesting that RGS8 upregulation in the SCA14 mouse model may have a protective role for Purkinje cells. In other SCAs, the decreased expression of RGS8 may contribute to increased mGluR1 signaling. Our findings support a critical role of mGluR1 signaling and its regulation by RGS8 in different types of SCAs.

### RGS8 Expression in the Cerebellum

We show that in the cerebellum RGS8 is specifically expressed in Purkinje cells starting after P7, and that Purkinje cells at P10 already express substantial amounts of RGS8 protein. This time sequence is in agreement with an earlier report about the developmental expression of RGS8 studied by *in situ*-hybridization. In this study, RGS8 mRNA was not detectable at P7, but substantial hybridization signal was found at P9 and later (Gold et al., 1997; Itoh et al., 2001; Saitoh and Odagiri, 2003). The beginning of the expression goes together with the expansion and maturation of Purkinje cell dendrites and the maturation of Purkinje cell electrophysiological properties (Armengol and Sotelo, 1991; McKay and Turner, 2005) and is also correlated with the maturation of mGluR1 expression and function in Purkinje cell dendrites (Ryo et al., 1993). This developmental expression profile is well compatible with a modulating role of RGS8 in mGluR1 signaling.

### Increase of RGS8 and mGluR1 Expression in the SCA14 PKCγ(S361G) Mouse Model

**FIGURE 7:**
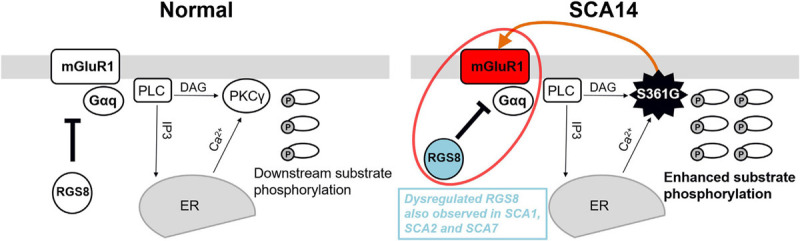
Model of the functional effect of RGS8 in SCA14. **Left panel:** mGluR1-PKCγ signaling pathway in the normal situation. mGluR1 activation via Gαq activates phosphoinositide phospholipase C (PLC) to produce inositol 1,4,5 trisphosphate (IP3) and diacylglycerol (DAG). IP3 binds to the IP3 receptor and calcium ions (Ca^2+^) are released from the endoplasmic reticulum (ER). The combination of DAG and Ca^2+^ activates PKCγ and induces the phosphorylation of downstream substrates. RGS8 is involved in the regulation of mGluR1 signaling. **Right panel:** In the SCA14 PKCγ(S361G) mutant with constitutively active kinase domain, the phosphorylation of PKC substrates is enhanced. PKCγ(S361G) can directly interact with mGluR1 (orange arrow) which may further promote mGluR1-PKCγ signaling. Increased expression of RGS8 may be a protective reaction for dysregulated mGluR1 signaling (red circle) and limits the mGluR1-PKCγ pathway activation in SCA14 PKCγ(S361G). Dysregulated RGS8 has been reported in several SCAs implicating dysregulation of the mGluR1 pathway within the pathogenesis of SCAs.

We show that RGS8 protein expression is increased in Purkinje cells with reduced dendritic expansion in organotypic slice cultures or dissociated cerebellar cultures from SCA14 PKCγ(S361G) mice. This increase is in contrast to the situation in SCA1, SCA2, and SCA7 where expression is decreased. The major change in PKCγ(S361G) Purkinje cells is a constitutive active kinase domain of PKCγ as reflected by the increased phosphorylation of PKC substrates in organotypic slice cultures from these mice (Figure 4A). Interestingly, this constitutive activation of PKCγ(S361G) also results in an increased expression of mGluR1 and Gαq/11 indicating elevated mGluR1 signaling in Purkinje cells. An mGluR1-PKCγ signaling cascade including mGluR1, Gαq, PLC, and PKCγ, has been shown to be important in cerebellar Purkinje cells. Genetic mouse models lacking either mGluR1, Gαq, PLC, or PKCγ all show similar phenotypes. Gαq regulates PLC which is activated and produces two intracellular messengers (inositol 1,4,5 trisphosphate) IP3 and diacylglycerol (DAG). IP3 binds to the IP3 receptor and induces the release of calcium. DAG together with increased calcium activates PKCγ (Kano et al., 1995, 1997; Offermanns et al., 1997). We have confirmed that the mutated PKCγ(S361G) can still bind to and interact with mGluR1 and does colocalize with mGluR1 in Purkinje cells. In the moment, we do not know whether the dysregulated in mGluR1 signaling is the result of this direct interaction. While the role of mGluR1 phosphorylation for LTD is well studied (for review see Kano et al., 2008), little is known about the effects of PKCγ-mediated phosphorylation of mGluR1 on its expression and long-term signaling. From our experiments, we cannot tell whether the increase of mGluR1 expression is a direct effect of increased mGluR1 phosphorylation by PKCγ(S361G) or is an indirect consequence of the chronically increased PKC activity present in the PKCγ(S361G) Purkinje cells. On the other hand, it seems very likely that the increased expression of RGS8 is due to the increase in mGluR1 signaling. This view is nicely compatible with the known function of RGS8 as a negative regulator of mGluR1 signaling. A model of the proposed RGS8 function in the SCA14 PKCγ(S361G) mouse is illustrated in Figure 7.

### Increased RGS8 Expression May Function as a Protective Modifier of Dysregulated mGluR1-PKC Signaling in Purkinje Cells of SCA14 PKCγ(S361G) Transgenic Mice

RGS8 is a member of the R4 subfamily of the regulator of G protein signaling (RGS) gene family and is shown to interact with Gαq/11. After binding, RGS8 is thought to accelerate the hydrolysis of GTP thereby limiting G protein activation (De Vries et al., 2000). Overshooting mGluR1 activation in Purkinje cells by DHPG is well known to cause a marked reduction of Purkinje cell dendritic tree development (Sirzen-Zelenskaya et al., 2006) similar to that found in PKCγ(S361G) Purkinje cells. We have now shown that overexpression of RGS8 in Purkinje cells does indeed protect the dendritic tree from DHPG induced reduction confirming the modulating role of RGS8 on mGluR1 signaling in Purkinje cells. The increased expression of RGS8 found in Purkinje cells from these mice appears to be a consequence of increased mGluR1 expression and is likely to be one of the molecular adjustments that does allow many Purkinje cells in SCA14 PKCγ(S361G) transgenic mice to develop a rather normal dendritic tree *in vivo* (Ji et al., 2014; Trzesniewski et al., 2019) despite constitutively increased PKC activity and changed mGluR1 expression.

### RGS8 and Disturbed mGluR1 Signaling Play Important Roles in the Development of SCAs

RGS8 is the only molecule with a known transcriptional dysregulation in four different mouse models of SCA, i.e., SCA1, SCA2, SCA7, and SCA14. Interestingly, the type of regulation appears to be different in the different disease types. In SCA1, RGS8 is downregulated (Gatchel et al., 2008; Ingram et al., 2016) and there is also evidence for a reduced activity of mGluR1 signaling (Notartomaso et al., 2013; Shuvaev et al., 2017) although in another study an increase in mGluR1 signaling was found (Power et al., 2016). The reduction of RGS8 expression in SCA1 has been attributed to regulation by microRNA (Rodriguez-Lebron et al., 2013). In contrast, in SCA2 there is evidence that RGS8 downregulation and the concomitant increase in mGluR1 signaling are critical for disease development and progression (Dansithong et al., 2015; Meera et al., 2016). In this model, the reduced expression of RGS8 directly leads to increased mGluR1 signaling which is causing Ca^2+^-dysregulation and cerebellar dysfunction. In SCA7, the contribution of mGluR1 signaling to disease development is less clear (Niewiadomska-Cimicka and Trottier, 2019), but the disruption of calcium homeostasis appears to be a critical aspect for Purkinje cell dysfunction and loss in the SCA7 mouse model. RGS8 was identified as one of the calcium regulatory genes with an altered expression in the SCA7 mouse model although its role for the observed disturbance in calcium regulation was not further explored in this study (Stoyas et al., 2020). Mutations in the mGluR1 gene itself also cause spinocerebellar ataxia, irrespective of whether these mutations are gain or loss of function mutations (Watson et al., 2017) and autoantibodies against mGluR1 are a common cause of autoimmune or paraneoplastic cerebellar ataxia (Joubert and Honnorat, 2019). In the SCA14 PKCγ(S361G) mice used in this study we have identified a dysregulated mGluR1 signaling which goes together with increased expression of RGS8. This suggests that the increase of RGS8 expression might be secondary to the elevation of mGluR1-PKC signaling as RGS8 is known to be a negative regulator of G-protein mediated signaling. We have shown that overexpression of RGS8 does indeed protect the Purkinje cell dendritic tree from DHPG induced mGluR1 stimulation. It is well known that mGluR1 stimulation is one of the major sources for a rise in intracellular calcium either by stimulating the IP3 receptor pathway or by Ca^2+^ entering through mGluR1-gated TRPC3 channels (Hartmann et al., 2008). Both ways of calcium rise upon mGluR1 stimulation require G-protein activation mediated by RGS8, pinpointing the crucial role of RGS8 for intracellular calcium regulation in Purkinje cells. As increasing evidence points toward a crucial role of Purkinje cell calcium regulation, in particular via the IP3 receptor pathway for the development of SCAs (Schorge et al., 2010; Brown and Loew, 2015; Shimobayashi and Kapfhammer, 2018), RGS8 emerges now as a major regulator of this pathway and the Purkinje cell calcium equilibrium making it an important determinant of pathogenesis of diverse SCAs.

## Conclusion

We have identified RGS8 as a gene being dysregulated in different mouse models of SCA and being specifically expressed in mouse cerebellar Purkinje cells. RGS8 upregulation in the SCA14 mouse model is related to dysregulated mGluR1-PKCγ signaling and we show that RGS8 overexpression protects Purkinje cell dendrites from the negative effects of mGluR1 activation. Our findings support a critical role of mGluR1 signaling and its regulation by RGS8 in the pathogenesis of different types of SCAs.

## Data Availability Statement

The raw data supporting the conclusions of this article will be made available by the authors, without undue reservation.

## Ethics Statement

The animal study was reviewed and approved by the Veterinary Office of the Canton of Basel.

## Author Contributions

Q-WW and JK conceived the idea for the study and wrote the manuscript. Q-WW conducted the experiments and data analyses. JK supervised the study. Both authors were involved in discussions on the final manuscript.

## Conflict of Interest

The authors declare that the research was conducted in the absence of any commercial or financial relationships that could be construed as a potential conflict of interest.
