# Supplementary material for: Modulation of Increased mGluR1 Signaling by RGS8 Protects Purkinje Cells From Dendritic Reduction and Could Be a Common Mechanism in Diverse Forms of Spinocerebellar Ataxia
Source: Front Cell Dev Biol. 2021 Jan 21;8:569889. doi: 10.3389/fcell.2020.569889 (PMC7858651; doi:10.3389/fcell.2020.569889)
Supplement: Supplementary file 1 [file Presentation_1.pdf]

Supplementary Material

1 Supplementary Figure 1

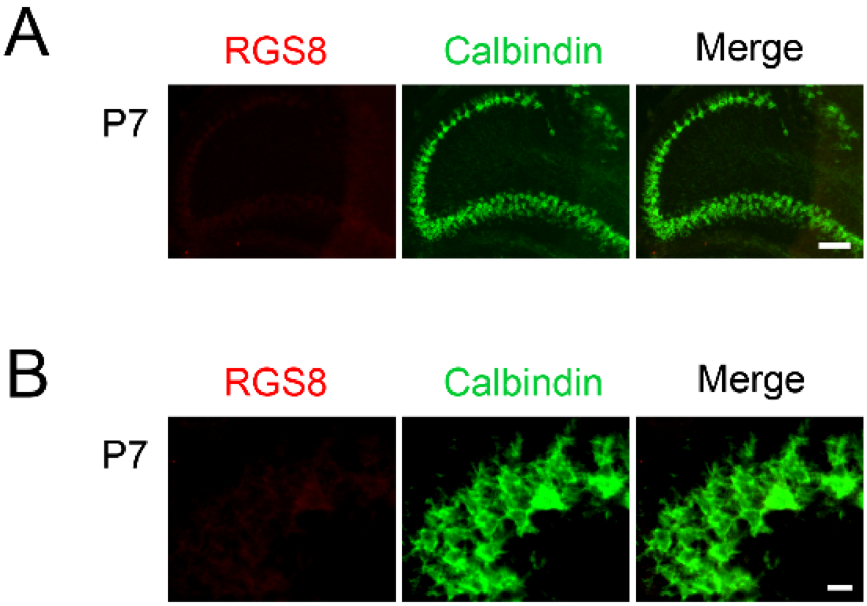

Supplementary Figure 1. (A) RGS8 immunoreactivity (red signal) was not obviously observed in cerebellar Purkinje cells (identified by anti-Calbindin, green) at P7. Scale bar is 100 µm. (B) Viewed at higher magnification, RGS8 was not present or little expressed in Purkinje cells at P7. Scale bar is 20 µm.

2 Supplementary Figure 2

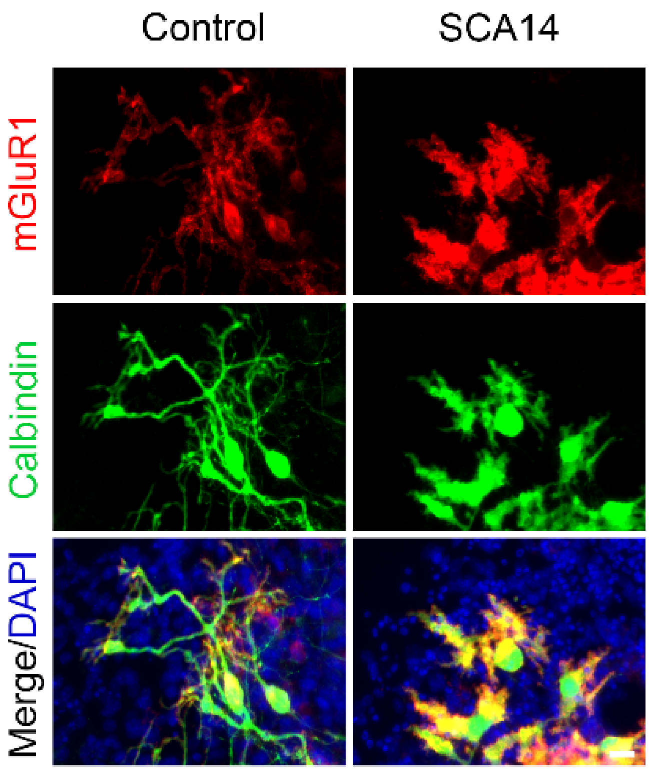

Supplementary Figure 2. mGluR1 immunoreactivity (red signal) was present in Purkinje cells in cerebellar slice cultures from SCA14 PKCγ(S361G) mice and littermate controls. Purkinje cells were identified by anti-calbindin staining (green). Cell nuclei were stained with DAPI (blue). Scale bar is 20 µm.

3 Supplementary Figure 3

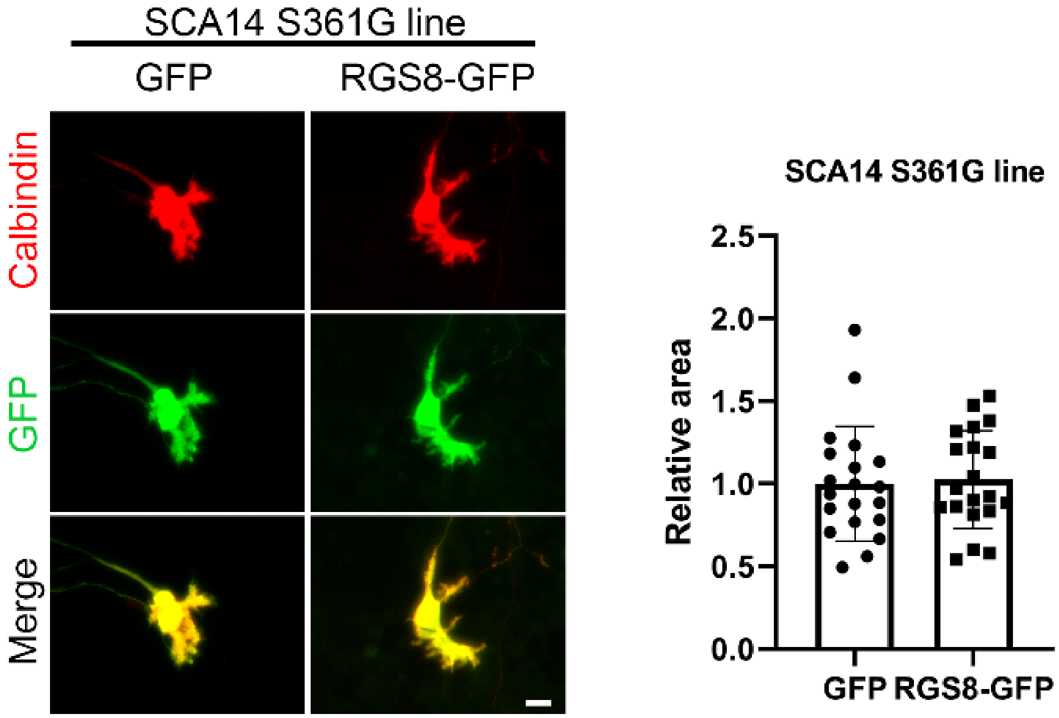

Supplementary Figure 3. Purkinje cell morphology of SCA14 mouse line was not significantly rescued after transfection of RGS8-GFP. Representative images of Purkinje cells after GFP or RGS8-GFP transfection. The mean values of the Purkinje cell dendritic area were measured in four independent culture wells. GFP transfection 1.000 ± 0.346 vs RGS8-GFP transfection 1.024 ± 0.296; n = 20 cells, P = 0.6395 in the two-tailed Mann-Whitney test. Data are expressed as mean ± SD. Scale bar is 20 µm.
